# miR‐223 improves intestinal inflammation through inhibiting the IL‐6/STAT3 signaling pathway in dextran sodium sulfate‐induced experimental colitis

**DOI:** 10.1002/iid3.395

**Published:** 2020-12-17

**Authors:** Juanjuan Zhang, Chenyang Wang, Zhen Guo, Binlin Da, Weiming Zhu, Qiurong Li

**Affiliations:** ^1^ Research Institute of General Surgery, Jinling Hospital Nanjing Medical University Nanjing Jiangsu China; ^2^ Research Institute of General Surgery Jinling Hospital Nanjing Jiangsu China

**Keywords:** cytokines, dextran sodium sulfate‐induced colitis, IL‐6/STAT3 signaling pathway, inflammatory bowel disease, miR‐223

## Abstract

**Introduction:**

The pathogenesis of inflammatory bowel disease (IBD) has not yet been clarified and is closely related to several pro‐inflammatory factors. MicroRNA‐233 (miR‐223) might be involved in the development of IBD; however, the mechanism underlying its pathogenesis is unclear. In this study, we attempted to determine the role of miR‐223 in dextran sodium sulfate (DSS)‐induced colitis and explore the involvement of the IL‐6/STAT3 pathway in the development of intestinal mucosal inflammation.

**Materials and Methods:**

Except control (WT) group, male C57BL/6 mice were provided DSS, then treated for with miR‐223 agomir or antagomir including DSS group, DSS + miR‐223 agomir (DSS + A) group, and DSS + miR‐223 antagomir (DSS + AN) group. The colitis symptoms were observed, the disease activity index (DAI) score were recorded daily, and colonic inflammation was evaluated by histopathological scoring. The expression of myeloperoxidase (MPO), cytokines and IL‐6/STAT3 pathway‐related proteins were measured.

**Results:**

miR‐223 expression in the terminal ileum and colon was increased in the DSS group compared with the WT group. Colitis symptoms were significantly alleviated in the DSS + A group and exacerbated in the DSS + AN group after administration of the miR‐223 agomir and antagomir, respectively. MPO, tumor necrosis factor‐α, IL‐6, and IL‐17 were decreased and IL‐10 was increased in the DSS + A group, but these changes were reversed in the DSS + AN group. Gp130, p‐STAT3, Bcl‐2, and Bcl‐xl in the colon declined in the DSS + A group, but these levels increased in the DSS + AN group.

**Conclusions:**

The upregulation of miR‐223 by agomir administration alleviated colonic inflammation in a DSS‐induced colitis model, which was likely mediated by inhibiting the production of pro‐inflammatory cytokines via the IL‐6/STAT3 signaling pathway. These findings provide evidence that miR‐223 might have potential therapeutic implications in IBD.

## 1 INTRODUCTION

Inflammatory bowel disease (IBD) is an immune‐mediated digestive tract inflammatory disease that includes Crohn's disease (CD) and ulcerative colitis (UC). IBD is a lifelong disease characterized by recurrent seizures accompanied by complications, such as perforation, strictures, fistula, or bleeding. The pathogenesis of IBD has not yet been clarified and is correlated with complex interactions between susceptibility genes, environmental factors, immunity, and inflammation.^1^

MicroRNA‐233 (miR‐223) is a crucial regulator of innate immunity, including myeloid differentiation and the function of neutrophils and macrophages.^2^ miR‐223 is highly expressed in neutrophils and is effective in limiting the activation and function of neutrophils in inflammatory diseases.^2^ Aberrant immune activation is a key trigger in the development of chronic mucosal inflammation in IBD.^3^ Recently, miR‐223 has been shown to participate in the regulation of the immune response and the production of cytokines in the pathogenesis of IBD.^4^, ^5^, ^6^, ^7^, ^8^ Several studies have reported the abnormal expression of miR‐223 in the circulation, intestinal mucosa, and feces in IBD.^9^, ^10^, ^11^, ^12^ Therefore, miR‐223 could be a biomarker of IBD to assess disease activity, suggesting that miR‐223 is a therapeutic target in IBD.

Our previous study showed that miR‐223 in the serum and intestinal mucosa were increased and correlated with IL‐6 expression in patients with CD. IL‐6/STAT3 is an important signaling pathway involved in the development of IBD. This study explored the potential role of miR‐223 in dextran sodium sulfate (DSS)‐induced colitis and its involvement in the IL‐6/STAT3 pathway during the pathogenesis of colitis.

## 2 MATERIALS AND METHODS

### 2.1 Mice and groups

Thirty‐six male C57BL/6 mice (6–8 weeks old) were obtained from the Animal Research Center of Jinling Hospital. All mice were maintained in specific pathogen free conditions, ensuring indoor ventilation and constant temperature and humidity. The mice received humane care in accordance with the law regarding the protection and control of animals in China. The mice were divided into six groups (*n* = 6): control (WT) group, DSS group, DSS + miR‐223 agomir (DSS + A) group, DSS + miR‐223 agomir negative control (DSS + A + NC) group, DSS + miR‐223 antagomir (DSS + AN) group, and DSS + miR‐223 antagomir negative control (DSS + AN + NC) group.

### 2.2 DSS‐induced colitis

Except for the WT group, the mice were provided 2.5% (wt/vol) DSS (MP Biomedicals) dissolved in drinking water ad libitum for 6 days. The DSS solution was replaced on alternate days. Body weight, stool consistency, and fecal blood were observed and recorded daily to calculate colitis disease activity index (DAI) scores. The DAI is based on body weight loss, stool consistency, and the presence or absence of fecal blood as clinical indicators of colitis. A score of 1–4 was given for each parameter (0: no weight loss, normal stool, no blood; 1: 1%–3% weight loss; 2: 3%–6% weight loss, loose stool, blood visible in stool; 3: 6%–9% weight loss; 4: > 9% weight loss, diarrhea, and gross bleeding), with ranges from 0 to 12. All mice were euthanized by CO_2_ asphyxiation on Day 7. The terminal ileum and colon were removed, colon length, and weight were measured as indications of colonic inflammation, and the colons were assessed by histological examination.

### 2.3 miR‐223 agomir and antagomir administration

miR‐223 agomirs and antagomirs (Genepharma) were used to enhance and inhibit miR‐223 expression in vivo, respectively. The miR‐223 agomir was a 22‐mer with the sequence 5′‐UGUCAGUUUGUCAAAUACCCCA‐3′ and 3′‐GGGUAUUUGACAAACUGACAUU‐5′, while the negative control sequence was a 22‐mer with the 5′‐UUCUCCGAACGUGUCACGUTT‐3′ and 3′‐ACGUGACACGUUCGGAGAATT‐5′. The miR‐223 antagomir was a 22‐mer with the sequence 5′‐UGGGGUAUUUGACAAACUGACA‐3′, while the negative control sequence was a 21‐mer with the 5′‐CAGUACUUUUGUGUAGUACAA‐3′. All substances were dissolved at a final concentration of 3 mg/ml in RNase‐free sterile phosphate‐buffered saline (PBS). Beginning 24 h after DSS administration, the mice were treated for three consecutive days with miR‐223 agomir (1.5 mg/kg/day, intraperitoneal injection), miR‐223 agomir negative control (1.5 mg/kg/day, intraperitoneal injection), miR‐223 antagomir (7.5 mg/kg/day, intraperitoneal injection), and miR‐223 antagomir negative control (7.5 mg/kg/day, intraperitoneal injection).

### 2.4 Histological assessment of colitis

The colonic tissues were excised, and processed as follows: the tissues were washed with ice‐cold PBS, fixed in 10% buffered formalin, embedded in paraffin, cut into 5‐µm sections, and stained with hematoxylin and eosin (H&E). The histology were scored blindly by two pathologists using previously described criteria: 0, no signs of inflammation; 1, very low level; 2, low level of leukocyte infiltration; 3, high level of leukocyte infiltration, high vascular density, thickening of the colon wall; and 4, transmural infiltration, loss of goblet cells, high vascular density, and thickening of the colon wall.^13^

### 2.5 Enzyme‐linked immunosorbent assay

Colon tissue samples were collected by enucleation, washed with PBS and stored at −80°C. Protein expression levels of myeloperoxidase (MPO), tumor necrosis factor‐α (TNF‐α), interleukin‐6 (IL‐6), IL‐10, and IL‐17 were determined using an enzyme‐linked immunosorbent assay (ELISA) kit (LiankeBio) according to the manufacturer's protocols.

### 2.6 Real‐time quantitative polymerase chain reaction

miRNAs in the terminal ileum and colon were isolated using a QIAGEN RNeasy mini kit (QIAGEN). The miRNA isolation procedure was performed according to the manufacturer's recommendations. For the quantification of mature miRNA, complementary DNA (cDNA) was generated using specific stem‑loop universal primers. Total RNA (1 µg) was reverse‐transcribed to cDNA by using the SCRIPT™ reverse transcription kit (RiboBio). Total RNA was extracted from colon tissue by using TRIzol reagent (Thermo Fisher Scientific), and the RNA was reverse‐transcribed into cDNA using a cDNA kit (Thermo Fisher Scientific). Bulge‐Loop™ miRNA real‐time quantitative polymerase chain reaction (RT‐qPCR) Primer Sets (One RT primer and a pair of qPCR primers for each set) specific for miR‐223‐3p were designed by RiboBio. The other primers used for RT‐PCR are shown in Table 1. The cDNA was quantified by a NanoDrop 2000 (Thermo Fisher Scientific). RT‐qPCR was performed using a 7500 Real‐Time PCR system (Applied Biosystems). All reactions were performed in triplicate. The relative expression levels were determined with the 2−ΔΔCt method.

**Table 1:** The primer sequences

GAPDH F	AGGAGCGAGACCCCACTAACA
GAPDH R	AGGGGGGCTAAGCAGTTGGT
IL‐6 mRNA F	GATGCTACCAAACTGGATATAATC
IL‐6 mRNA R	GGTCCTTAGCCACTCCTTCTGTG
TNF‐α mRNA F	GCATGATCCGCGACGTGGAA
TNF‐α mRNA R	AGATCCATGCCGTTGGCCAG
IL‐10 mRNA F	GGTCTGAGTGGGACTCAAGG
IL‐10 mRNA R	CGTGGCAATGATCTCAACAC
IL‐17 mRNA F	AAAGCTCAGCGTGTCCAAAC
IL‐17 mRNA R	TGAGCTTCCCAGATCACAGA
gp130 mRNA F	ATTTGTGTGCTGAAGGAGGC
gp130 mRNA R	AAAGGACAGGATGTTGCAGG
Bcl‐2 mRNA F	ATCTTCTCCTTCCAGCCTGA
Bcl‐2 mRNA R	ACCGAACTCAAAGAAGGCCA
Bcl‐xl mRNA F	CGACTTTCTCCDTCCTACAAGC
Bcl‐xl mRNA R	CTGCTCAAAGCTCTGATACG

### 2.7 Western blot analysis

Colonic tissues were lysed in radioimmunoprecipitation assay buffer (Sigma) containing a mixture of protease inhibitor cocktail kits (Thermo Fisher, USA) for 30 min, and then homogenized using an ultrasonic cell crushing apparatus (FCNCS Technology Laboratories, China). The proteins concentrations were determined using bicinchoninic acid protein assay kit (Thermo Fisher Scientific). The proteins were separated on 8% sodium dodecyl sulfate‐polyacrylamide gels. The proteins were subjected to electrophoresis and transferred onto polyvinylidene difluoride membranes (0.45 µm, Amersham Biosciences). The membranes were blocked with 5% nonfat dry milk at room temperature after transfer. Subsequently, the membranes were incubated overnight at 4°C with rabbit‐monoclonal anti‐STAT3 antibody (Abcam), anti‐STAT3 (phosphor Y705) antibody (Abcam), and anti‐GAPDH (Abcam) antibody. The horseradish peroxidase (Santa Cruz)‐conjugated secondary antibody (goat antirabbit antibody, Abcam) was added after washing with Tris buffered saline. The expression of STAT3 and p‐STAT3 was quantified relative to GAPDH expression with Image software (National Institutes of Health).

### 2.8 Statistical analyses

The experimental data are expressed as the mean ± *SD* and the statistical significance between two groups was determined by Student *t*‐test using SPSS software (version 22.0). A value of *p* < .05 was considered significant.

### 2.9 Ethics statement

All the experiments and procedures were performed with the approval of the Institutional Ethics Committee of Jinling Hospital.

## 3 RESULTS

### 3.1 The expression of miR‐223 in the terminal ileum and colon

**Figure 1:**
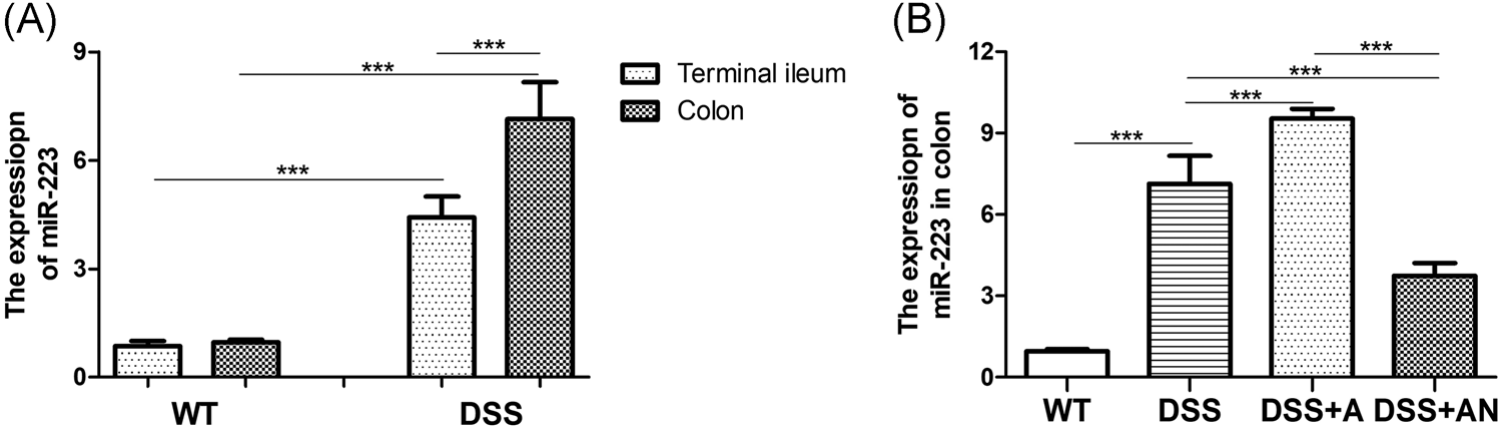
The expression of miR‐223 in terminal ileum and colon (*n* = 6/group). (A) The expression of miR‐223 in terminal ileum and colon of the DSS group compared with the WT group, (B) The expression of miR‐223 in colon, where ***p < .001. DSS, dextran sodium sulfate; miR, microRNA; WT, wild‐type

miR‐223 expression in the terminal ileum and colon in the DSS group was increased compared to that of the WT group (Figure 1A). In the DSS group, miR‐223 expression in the colon was higher than that in the terminal ileum (Figure 1A). Compared with those of the DSS group, colonic miR‐223 expression levels were increased in the DSS + A group and were decreased in the DSS + AN group (Figure 1B). There were significant differences in miR‐223 expression among the DSS, DSS + A, and DSS + AN groups.

### 3.2 General characteristics of experimental colitis

**Figure 2:**
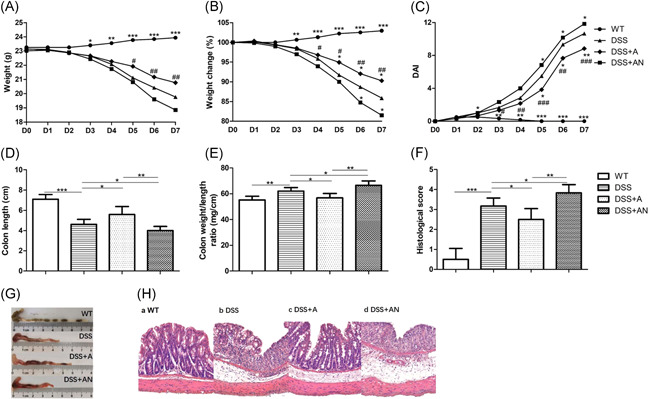
The effects of miR‐223 agomir and antagomir on DSS‐induced colitis (*n* = 6/group). (A) The changes of daily weight, (B)The changes of daily weight change, (C) The changes of daily DAI scores (where * indicates the comparison between DSS and WT, DSS + A, DSS + AN group, respectively, # indicates the comparison between DSS + A and DSS + AN group). (D) The changes of colon lengths. (E) The changes of colon weight/length ratios. (F) The changes of histological scores. (G) Representative macroscopic images of colons. (H) Representative micrographs of colon H&E (×200), where *p < .05, **p < .01, ***p < .001, #*p* < .05, ##*p* < .01, ###*p* < .001. DSS, dextran sodium sulfate; miR, microRNA; WT, wild‐type

In the DSS group, the absolute weight and weight change (Day X/Day 0) were decreased, and the DAI score gradually increased every day (Figure 2A‐C). The daily weights of mice in the DSS + A group were significantly higher than those of mice in the DSS + AN group beginning on Day 3 (Figure 2A). Compared with that of the DSS group, the daily weight change was higher in the DSS + A group beginning on Day 5 and was lower in the DSS + AN group beginning on Day 6 (Figure 2B). Bloody stool appeared later in the DSS + A group, but appeared earlier in the DSS + AN group than in the other groups. The DAI score decreased beginning on Day 5 in the DSS + A group, and increased beginning on Day 5 in the DSS + AN group (Figure 2C). There were significant differences in daily weights, weight changes, and DAI scores between the DSS + A and DSS + AN groups.

### 3.3 Morphological and histological changes in the colon

The length of colons was shortened, and the weight/length ratio of the colon and histological inflammatory score were increased in the DSS group compared with the WT group (Figure 2D‐H). Compared with that of the DSS group, the colon length was longer in the DSS + A group and was shorter in DSS + AN group, and the colon weight/length ratio was lower in the DSS + A group and higher in the DSS + AN group (Figures 2D,E and 2G). The histological inflammatory scores of H&E‐stained colon tissue decreased in the DSS + A group and increased in the DSS + AN group compared with the DSS group (Figures 2F and 2H). These differences between the DSS + A and DSS + AN groups were all significant.

### 3.4 The levels of MPO and cytokines

**Figure 3:**
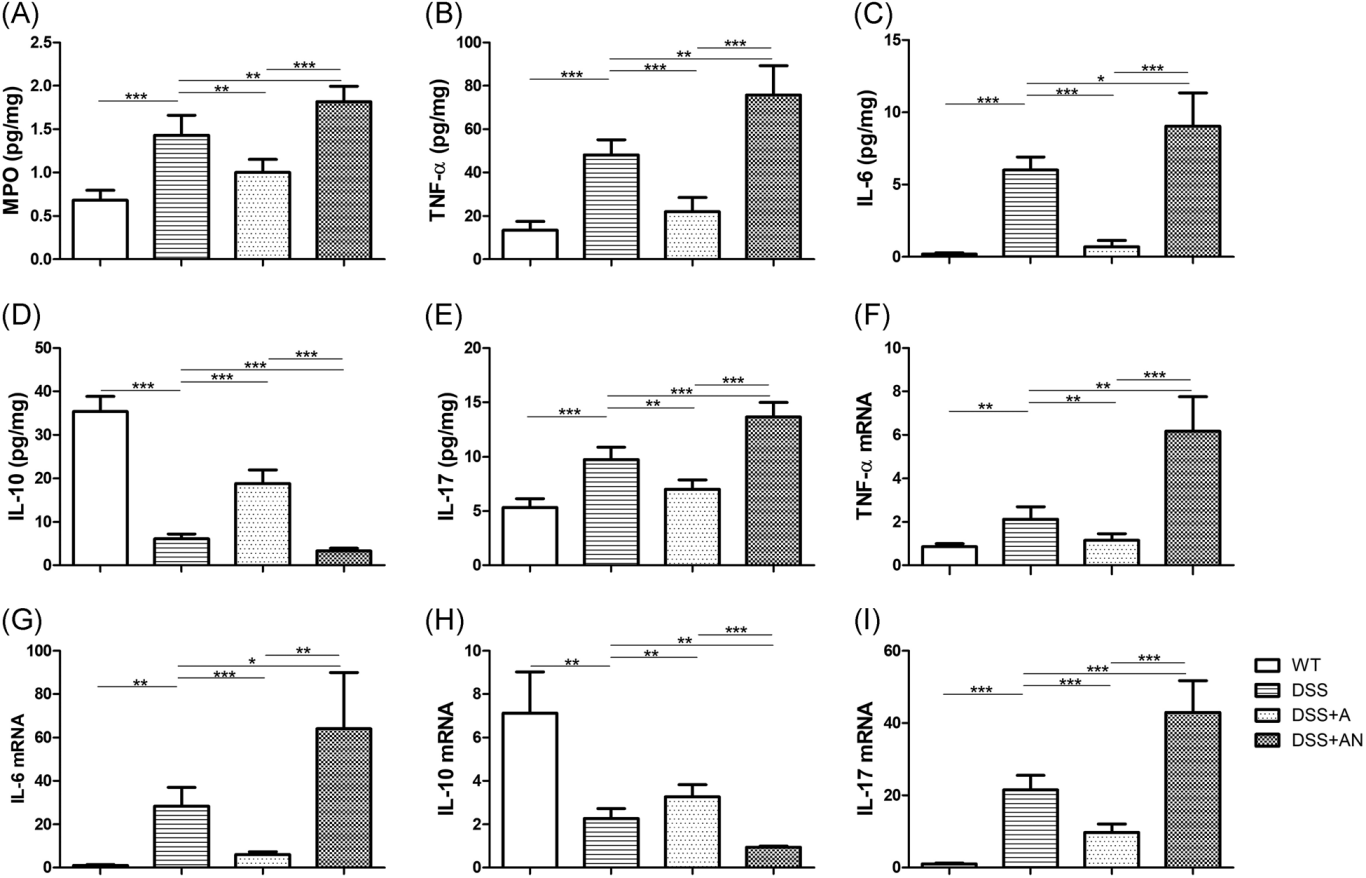
The effects of miR‐223 agomir and antagomir on MPO and protein and mRNA levels of cytokines in colon (*n* = 6/group). (A) The changes of MPO level. (B) The changes of TNF‐α level. (C) The changes of IL‐6 level. (D) The changes of IL‐10 level. (E) The changes of IL‐17 level. (F) The changes of TNF‐α mRNA level. (G) The changes of IL‐6 mRNA level. (H) The changes of IL‐10 mRNA level. (I) The changes of IL‐17 mRNA level, where * *p* < .05, **p < .01, ***p < .001. IL, IL, interleukin; miR, microRNA; MPO, myeloperoxidase; mRNA, messenger RNA; TNF‐α, tumor necrosis factor‐α

The expression of MPO, TNF‐α, IL‐6, IL‐10, and IL‐17 were measured by ELISA to detect the protein levels, and the expression of TNF‐α, IL‐6, IL‐10, and IL‐17 were also measured by RT‑qPCR to detect the mRNA levels. We found that the levels of MPO, TNF‐α, IL‐6, and IL‐17 in the colon were increased, and IL‐10 was decreased in the DSS group compared with the WT group. In the DSS + A group, the levels of MPO, TNF‐α, IL‐6, IL‐17 declined and IL‐10 was elevated compared with those of the DSS group. In contrast, MPO, TNF‐α, IL‐6, and IL‐17 were elevated and IL‐10 was decreased in the DSS + AN group. There were significant differences in the levels of MPO and cytokines between the DSS + A and DSS + AN groups (Figure 3).

### 3.5 Changes in the IL‐6/STAT3 signaling pathway

**Figure 4:**
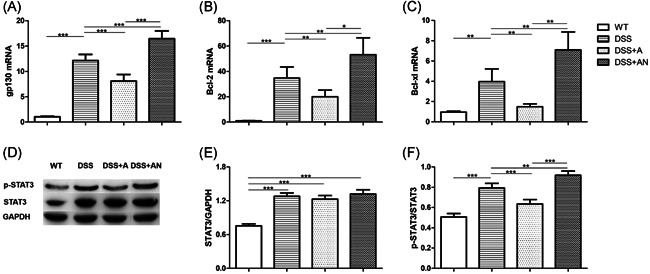
The effects of miR‐223 agomir and antagomir on IL‐6/STAT3 pathway (*n* = 6/group). (A) The changes of gp130 mRNA level. (B) The changes of Bcl‐2 mRNA level. (C) The changes of Bcl‐xl mRNA level. (D) The expression changes of STAT3 and p‐STAT3. (E) The changes of STAT3/GAPDH. (F) The changes of p‐STAT3/STAT3, where *p < .05, **p < .01, ***p < .001. IL, interleukin; miR, microRNA; mRNA, messenger RNA

The expression of gp130, Bcl‐2, and Bcl‐xl were measured by RT‑qPCR, and the expression of STAT3 and p‐STAT3 were measured by western blot analysis. We found that the expression levels of gp130, STAT3, p‐STAT3, Bcl‐2, and Bcl‐xl in the colon were increased in the DSS group compared with the WT group (Figure 4). Compared with those of the DSS group, gp130, Bcl‐2, and Bcl‐xl decreased in the DSS + A group and increased in the DSS + AN group (Figure 4A‐C). The expression of STAT3 was higher in the DSS group, DSS + A group, and DSS + AN group than in the WT group, and there had no differences among the DSS, DSS + A, and DSS + AN groups (Figure 4D,E). P‐STAT3 expression declined in the DSS + A group and increased in the DSS + AN group compared with the DSS group (Figures 4D and 4F). There were significant differences in gp130, Bcl‐2, Bcl‐xl, and p‐STAT3 levels between the DSS + A and DSS + AN groups (Figure 4).

## 4 DISCUSSION

**Figure 5:**
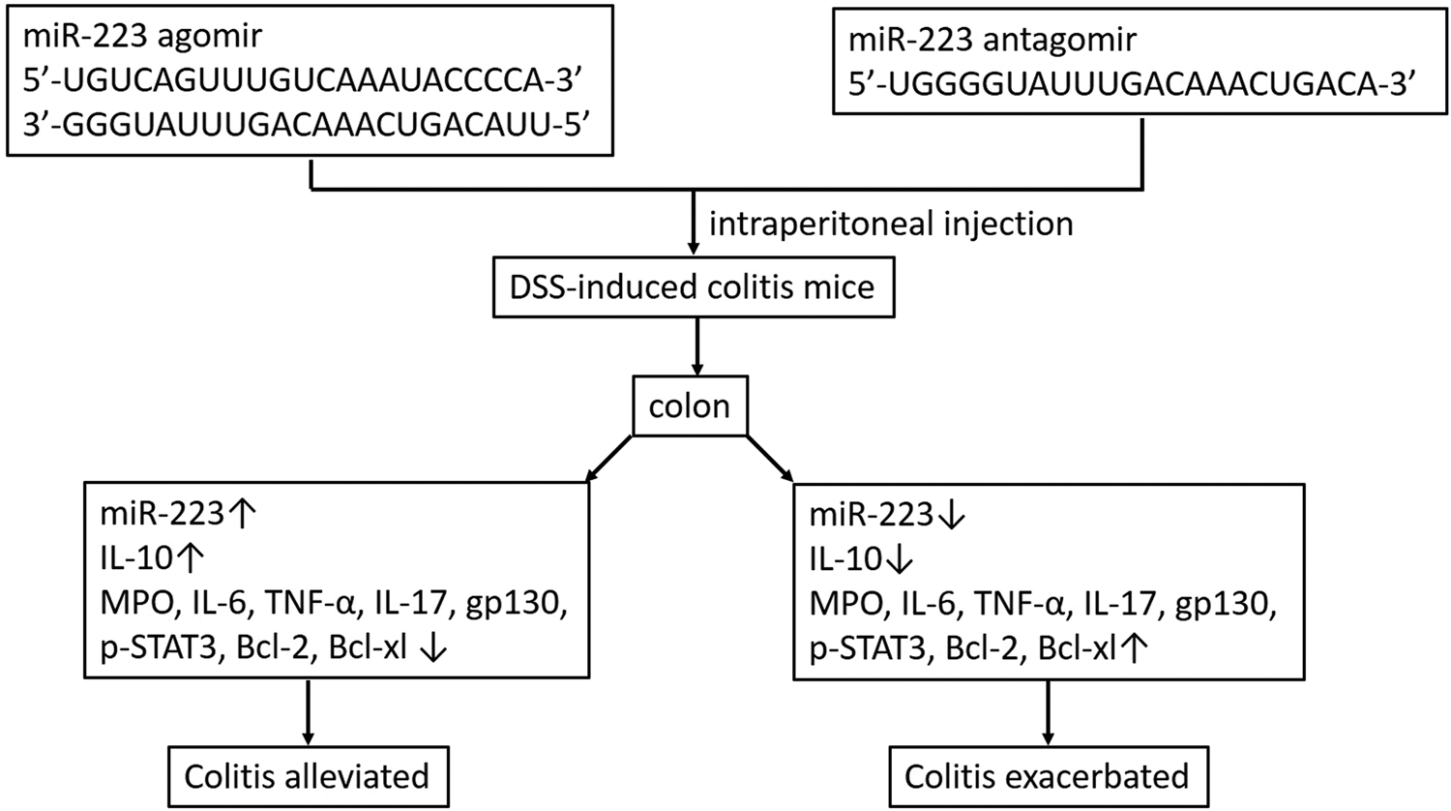
The effects of miR‐223 agomir and antagomir on DSS‐induced colitis mice. DSS, dextran sodium sulfate; miR, microRNA

In this study, we demonstrated that miR‐223 expression was upregulated in the DSS‐induced mice compared with WT mice. We also found that the upregulation of miR‐223 by agomir administration alleviated colitis, and downregulation of miR‐223 by antagomir administration exacerbated colitis (Figure 5). These findings suggested that miR‐223 might play a protective role in regulation of colonic inflammation through inhibiting the IL‐6/STAT3 signaling pathway in the DSS‐induced colitis model.

miR‐223 is a key modulator of the regulation of myeloid cells, especially the differentiation and activation of neutrophils and macrophages.^2^ Neutrophils are crucial in the development and modulation of mucosal inflammation.^14^ miR‐223 is highly expressed in neutrophils and is involved in the activation and function of neutrophils in inflammatory diseases.^2^ miR‐223 was participated in the regulation of neutrophilic airway inflammation through inhibiting the activation of NLRP3/IL‐1β signaling pathway in the asthma model.^15^ During acute respiratory distress syndrome (ARDS), miR‐223 is secreted in microvesicles from neutrophils that are recruited from the vasculature to sites of pulmonary inflammation and tissue injury and then shuttled to alveolar type II epithelial cells (ATII) to confer lung protection.^16^ Zhou et al.^17^ reported that neutrophilic inflammation (wound response) was augmented in miR‐223‐deficient zebrafish through elevated activation of the canonical nuclear factor κB (NF‐κB) pathway. Therefore, miR‐223 plays essential role to limit the activation and function of neutrophil in inflammatory diseases. Recent studies have demonstrated the abnormal expression of miR‐223 in patients with IBD and experimental colitis mice.^4^, ^5^, ^6^, ^7^, ^8^, ^9^, ^10^, ^11^, ^12^ In this study, we found that the expression of miR‐223 in the terminal ileum and colon was increased in DSS‐induced colitis mice. These results were similar to those of previous reports.

The pathogenesis of IBD is not completely clear and is related to dysregulated epithelial barrier function, increased bacterial translocation, and an aberrant immune response.^18^ miR‐223 not only functions as a key modulator for the innate immunity, but also affects adaptive immunity, and immune dysregulation has been shown to contribute to IBD.^2^ It has been reported that miR‐223 is involved in the pathogenesis of IBD by regulating various immune cells and signaling pathways.^4^, ^5^, ^6^, ^7^, ^8^ Furthermore, miR‐223 from human mast cells‐1 (HMCs‐1) inhibits CLDN8 expression to destroy intestinal barrier function in IBD.^19^

The role of miR‐223 in the pathogenesis of IBD is still controversial. Zhou et al.^8^ showed that miR‐223^−/y^ mice developed more severe colitis in the DSS‐induced colitis model, and concluded that miR‐223 regulated DCs and macrophages by directly targeting C/EBPβ to maintain intestinal homeostasis. Neudecker et al.^5^ also reported that miR‐223^−/y^ mice exhibited exacerbated experimental colitis with elevated NLRP3 and IL‐1β levels, and nanoparticle‐mediated overexpression of miR‐223 attenuated experimental colitis.^5^ Therefore, the researchers determined that miR‐223 was an anti‐inflammatory factor. In the present study, miR‐223 expression in the colon was significantly upregulated and downregulated by the miR‐223 agomir and antagomir, respectively. We also found that the clinical signs of experimental colitis were improved, MPO and pro‐inflammatory cytokines (TNF‐α, IL‐6, and IL‐17) were downregulated and anti‐inflammatory cytokine (IL‐10) were upregulated after administration of the miR‐223 agomir, and these changes were reversed after administration of the miR‐223 antagomir. These results showed that miR‐223 played an anti‐inflammatory role in DSS‐induced colitis. Interestingly, miR‐223 has been reported to be pro‐inflammatory by other studies. Wang et al.^7^ identified miR‐223 as a mediator of the crosstalk between the IL‐23 signaling pathway and CLDN8 in the development of IBD, and the inhibition of miR‐223 alleviated TNBS‐induced colitis. Kim et al.^4^ confirmed that miR‐223 negatively regulated FOXO3a to enhance NF‐κB signaling and promote the production of pro‐inflammatory cytokines.

miR‐223 has been confirmed to participate in the IL‐6/STAT3 pathway to regulate inflammatory cytokines.^20^, ^21^ Chen et al.^20^ suggested that IL‐6/miR‐223/STAT3 formed a positive feedback loop to promote the pathogenesis of inflammatory diseases. Wu et al.^21^ also found that miR‐223 formed a complex regulatory network with pro‐inflammatory factors and signaling pathways in adipose stem cells stimulated by lipopolysaccharide (LPS). However, whether miR‐223 is involved in IBD through the IL‐6/STAT3 pathway is unclear. In the present study, we explored the mechanism of miR‐223 in IBD, focusing on the IL‐6/STAT3 pathway. After administration of the miR‐223 agomir, the expression of miR‐223 was increased, and the levels of IL‐6, gp130, p‐STAT3, and antiapoptotic genes Bcl‐2 and Bcl‐x1 were decreased. The miR‐223 antagomir showed the opposite effects. These data demonstrated that miR‐223 might inhibit the IL‐6/STAT3 signaling pathway in DSS‐induced colitis.

In conclusion, this study demonstrated that upregulation of miR‐223 could attenuate the clinical signs of experimental colitis and colonic inflammation in a DSS‐induced colitis model. Furthermore, the anti‐inflammatory roles of miR‐223 are probably attributed to profound decreases in pro‐inflammatory cytokines through inhibition of the IL‐6/STAT3 signaling pathway. The data provide novel insights into the molecular mechanisms of IBD, and alterations in the miR‐223/IL‐6/STAT3 signaling pathway may be involved in the inflammatory response and contribute to the pathogenesis of chronic inflammatory disease. We also add evidence suggesting that miR‐223 could be considered a therapeutic target for the treatment of IBD. Future studies are needed to identify the anti‐inflammatory role of miR‐223 and its therapeutic potential in IBD patients.

## AUTHOR CONTRIBUTIONS

Juanjuan Zhang, Zhen Guo, Weiming Zhu, and Qiurong Li conceived and designed the study; Juanjuan Zhang, Chenyang Wang, and Binlin Da performed the experiments; Juanjuan Zhang and Chenyang Wang performed data analysis and wrote the article; Weiming Zhu and Qiurong Li reviewed and edited the manuscript. All authors have read and approved the final manuscript.

## CONFLICTS OF INTEREST

The authors declare that there are no conflicts of interest.

## Data Availability

The data that support the findings of this study are available from the corresponding author upon reasonable request.
