# Correction to “miR‐223 Improves Intestinal Inflammation Through Inhibiting the IL‐6/STAT3 Signaling Pathway in Dextran Sodium Sulfate‐Induced Experimental Colitis”

**DOI:** 10.1002/iid3.70209

**Published:** 2025-05-21

J. Zhang, C. Wang, Z. Guo, B. Da, W. Zhu and Q. Li, “miR‐223 Improves Intestinal Inflammation Through Inhibiting the IL‐6/STAT3 Signaling Pathway in Dextran Sodium Sulfate‐Induced Experimental Colitis,” *Immunity, Inflammation and Disease* 9, no. 1 (2021): 319‐327, https://doi.org/10.1002/iid3.395.

The gel in Figure 4D was cut following the reviewer's suggestion during peer review. The uncut, annotated gels have been provided as a supplemental figure. The authors confirm that all the experimental results and corresponding conclusions mentioned in the paper remain unaffected. Supplemental Figure is shown as follows.

Supplemental Figure


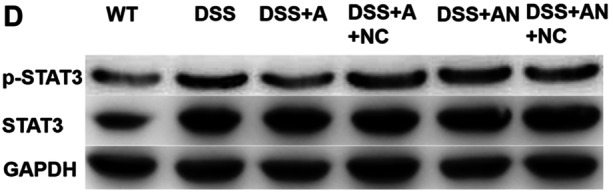


The authors apologize for this error.

